# Major depressive disorder is associated with risky driving behavior among older adults: A naturalistic study

**DOI:** 10.1093/geroni/igaf122.373

**Published:** 2025-12-31

**Authors:** David Carr, Ganesh Babulal

**Affiliations:** Washington University in St. Louis, St Louis, Missouri, United States; Washington University School of Medicine in St. Louis, SAINT LOUIS, Missouri, United States

## Abstract

We examined the impact of Major Depressive Disorder (MDD) on naturalistic driving behavior among older drivers. We hypothesized that cases with MDD would exhibit riskier driving behaviors, visit fewer destinations, and have fewer trips compared to nondepressed controls. A prospective, longitudinal, case-control study was conducted with 395 older adults (≥65 years). The sample included 85 cases with MDD and 310 controls. Neurology, clinical, and neuropsychological tests were collected annually. Daily driving behavior was recorded using a datalogger. Linear mixed models with propensity score weighting compared driving behaviors between groups, adjusting for sociodemographic variables, and medication use. MDD subjects exhibited higher numbers of speeding events (mean = 2.34 vs. 1.67, p<.001) and more hours driven per month (mean = 24.8 vs. 21.2, p<.001) compared to controls at baseline. Longitudinal analysis showed MDD subjects had an increase in hard braking (β = 0.27, p<.01) and hard cornering events (β = 0.21, p<.05) per trip, greater distances driven from home (β = 2.45 miles, p<.01), more unique destinations visited (β = 1.85, p<.05), and higher random entropy (β = 0.13, p<.05) over time. Post-hoc analysis adjusting for antidepressants and total medication use did not alter the results. Older adults with MDD exhibit distinct and riskier driving behaviors compared to non-depressed controls, with increased hard braking, cornering, and unpredictability in driving patterns over time. Routine depression screening and tailored interventions are essential for enhancing driving safety and maintaining independence among older adults with MDD. Comprehensive care approaches addressing both mental and physical health are crucial for this vulnerable population.

